# The Mechanisms of Cadmium Stress Mitigation by Fungal Endophytes from Maize Grains

**DOI:** 10.3390/jof10080581

**Published:** 2024-08-16

**Authors:** Muhammad Awais, Yingying Xiang, Dian Yang, Yibin Lai, Fenglian Cai, Naila Shah, Majid Khan, Haiyan Li

**Affiliations:** 1 Faculty of Environmental Science and Engineering, Medical School, Kunming University of Science and Technology, Kunming 650500, China; 2 The Affiliated Yanan Hospital of Kunming Medical University, Kunming 650051, China; 3 Department of Botany, Gardan Campus, Abdul Wali Khan University Mardan, Mardan 23200, Pakistan; 4 Institute of Cotton Research, Chinese Academy of Agriculture Sciences, Anyang 455000, China

**Keywords:** *Zea mays*, seed endophytes, plant growth promotion, Cd tolerance, pot experiment, corn grains, heavy metal contamination

## Abstract

Maize is a crucial staple crop that ensures global food security by supplying essential nutrients. However, heavy metal (HM) contamination inhibits maize growth, reduces output, and affects food security. Some endophytic fungi (EFs) in maize seeds have the potential to enhance growth and increase dry biomass, offering a solution to mitigate the negative effect of HM contamination. Using these functional EFs could help maintain crop production and ensure food safety in HM-contaminated areas. In the present study, the diversity of EFs in corn grains from various HM-contaminated areas in China was studied through culture-dependent and culture-independent methods. We tested the plant growth-promoting (PGP) traits of several dominant culturable isolates and evaluated the growth-promoting effects of these twenty-one isolates through pot experiments. Both studies showed that HM contamination increased the diversity and richness of corn grain EFs and affected the most dominant endophytes. *Nigrospora* and *Fusarium* were the most prevalent culturable endophytes in HM-contaminated areas. Conversely, *Cladosporium* spp. were the most isolated endophytes in non-contaminated areas. Different from this, *Saccharomycopsis* and *Fusarium* were the dominant EFs in HM-contaminated sites, while *Neofusicoccum* and *Sarocladium* were dominant in non-contaminated sites, according to a culture-independent analysis. PGP trait tests indicated that 70% of the tested isolates (forty-two) exhibited phosphorus solubilization, IAA production, or siderophore production activity. Specifically, 90% of the tested isolates from HM-contaminated sites showed better PGP results than 45% of the isolates from non-contaminated sites. The benefit of the twenty-one isolates on host plant growth was further studied through pot experiments, which showed that all the isolates could improve host plant growth. Among them, strains derived from HM-contaminated sites, including AK18 (*Nigrospora*), AK32 (*Beauveria*), SD93 (*Gibberellia*), and SD64 (*Fusarium*), had notable effects on enhancing the dry biomass of shoots and roots of maize under Cd stress. We speculate that the higher ratio of PGP EFs in corn grains from HM-contaminated areas may explain their competitiveness in such extreme environments. *Fusarium* and *Cladosporium* isolates show high PGP properties, but they can also be phytopathogenic. Therefore, it is essential to evaluate their pathogenic properties and safety for crops before considering their practical use in agriculture.

## 1. Introduction

Maize is widely cultivated in various ecological zones and is an important staple crop that helps ensure global food security. It provides essential nutrients for human consumption, serves as a predominant animal feed source, and has various industrial applications [1,2]. However, the long-term sustainability of maize farming is seriously threatened by the spread of environmental pollutants, especially those caused by hazardous compounds such as cadmium (Cd), lead (Pb,) and arsenic (As) [3,4,5]. Human activities such as mining, industrial pollution, and irrigation with tainted water can contaminate agricultural soils [3,6,7]. The persistence of heavy metals (HMs) in the food chain increases the possibility of their bioaccumulation and poses severe threats to food security and public health. Furthermore, this significant level of HM inhibits plant growth and reduces crop output [8,9,10,11].

The symbiotic relationship between plants and their microbiomes, particularly endophytic fungi (EFs), is critical for allowing plants to resist environmental stressors such as high levels of HM toxicity [12,13,14]. EFs are nonpathogenic microorganisms that live in plant tissues and provide various benefits to host plants, including increased nutrient uptake [15], disease resistance [16], and stress tolerance [17]. Some EFs in plants have the potential to enhance growth and increase dry biomass, offering a solution to mitigate the negative effect of HM contamination [17]. When exposed to metal stress, these fungi improve plant growth by generating phytohormones, solubilizing phosphates, and synthesizing siderophores [18,19].

Recent research has sought to explore the complex interactions within the plant microbiome, emphasizing the numerous microbial populations that inhabit different plant parts at different phases of development [20,21]. Seeds serve as small-scale ecosystems that harbor diverse microorganisms that potentially impact plant development and reproductive processes [21,22,23]. Gaining knowledge about these microbiomes is crucial for managing diseases, ensuring food safety, and discovering microorganisms that can aid in removing HMs from edible plant parts [24].

In Asian countries, maize is cultivated in HM-contaminated areas, and the HM concentration in corn grains exceeds the food standard of HM, posing risks to human health [25,26]. To ensure the safe production of corn in these areas, relationships between the EF of corn grains and the environment contaminated with HMs are essential for improving the resilience of maize crops to environmental stresses [27,28]. Nevertheless, there is a substantial lack of understanding regarding how seed endophytes in maize respond to HM pollution and their contribution to the host plant’s ability to tolerate and accumulate HMs [2]. This work seeks to close this disparity by examining the seed EF population of maize from HM-contaminated and non-contaminated areas, using culture-dependent and culture-independent approaches. In addition, the dominant EF plant growth-promoting (PGP) properties were evaluated.

## 2. Materials and Methods

### 2.1. Sampling Sites and Seed Collection

The following sites located in Yunnan Province, Southwest China, were chosen for study: Ayka (AK), Sanduoduo (SD), and Dali (DL). There were notable differences in the HM concentration in the soils between the chosen sites. AK was a highly contaminated area, SD was a modestly contaminated area, and DL was a non-contaminated area (Table 1).

Fresh corn grains were collected from each location. The samples were meticulously packed within sterile plastic bags to avoid any external contamination. After collection, the seeds were transferred to the laboratory in a controlled environment to guarantee the preservation of their integrity.

### 2.2. Seed Treatment

To remove any potential contamination, the corn seeds were carefully surface-sterilized according to [29]. The seeds were immersed in 75% ethanol for two minutes and then washed with sterile distilled water. After that, 5% sodium hypochlorite was applied for another two minutes, followed by three to five rinses with sterile distilled water. The surface-sterilized seeds were dried on sterilized filter paper and then used to make imprints on Petri dishes with potato dextrose agar (PDA). The absence of fungal growth within these imprints showed that the surface microorganisms had been successfully removed. The sterilized seeds were divided into two parts: one was preserved at −80 °C for high-throughput sequencing, and the other was preserved for EF isolation.

### 2.3. Culture-Dependent Analysis

Before EF isolation, each sterilized seed was cut into two pieces with sterile blades. Then, the pieces were placed onto the surface of PDA plates supplemented with 0.5 g/L streptomycin sulfate and incubated at 25 °C for EF growth. Five pieces were placed in each Petri dish. A total of 375 surface-sterilized seed pieces, 125 from each site (AK, SD, and DL), were investigated. The plates were incubated at 25 °C for 45 days and checked every other day. The fungi grown from the seeds were purified and kept on PDA slants [30,31].

EFs were identified based on morphological characteristics and internal transcribed spacer (ITS) region analysis [32]. For ITS region analysis, the CTAB-extracted genomic DNA from fungal isolates was amplified using the primers ITS1 and ITS4 [31]. Following purification with the Cyclepure Kit (Bioteke, Beijing, China), the PCR products were then delivered to Sangon Biotech Co., Ltd., for sequencing. The acquired sequences were uploaded to the GenBank database (https://www.ncbi.nlm.nih.gov/, accessed on 17 March 2024) for comparison with published sequences via BLAST.

The EF community was assessed using quantitative techniques. The colonization frequency (CF) was estimated by dividing the number of endophyte-colonized segments by the total number of incubation segments. The relative species frequency (RF) represented the proportion of isolates from a specific species to the total number of isolates [33]. Diversity indices were calculated to assess the richness and evenness of the EF community. The Shannon diversity index (H0) was calculated as H0 = −∑k i = 1 Pi × ln Pi, where k is the total number of fungal species and Pi is the fraction of individuals contributed by species I to the total [34]. Pielou’s evenness index, J = H0/log (S), was used to determine values of evenness, where S represents the number of species present (species richness) [35].

### 2.4. Culture-Independent Analysis

A comprehensive strategy was used to study the culture-independent EF community. The surface-sterilized seeds were thoroughly mixed in a sterile mortar using liquid nitrogen. Total genomic DNA was extracted from 0.2 g of this homogenized powder using the MoBio PowerSoil^®^ DNA Isolation Kit (MO BIO Laboratories, Inc., Carlsbad, CA, USA). After confirmation by electrophoresis on a 1.5% (*w*/*v*) agarose gel, the extracted DNA was carefully preserved at −20 °C in preparation for further examination.

The primers ITS1F (5′-CTTGGTCATTTAGAGGAAGTAA-3′) and ITS2R (5′-GCTGCGTTCTTCATCGATGC-3′) were used to amplify the fungal ITS1 gene region. The final amplification product was approximately 435 bp in length. The PCR parameters were as follows: predenaturation at 98 °C for 3 min, denaturation at 94 °C for 45 s, annealing at 55 °C for 45 s, extension at 72 °C for 45 s, a total of 30 cycles, and a final extension at 72 °C for 7 min. The recovered PCR products were purified using the AxyPrep DNA Gel Extraction Kit (Axygen Biosciences, Union City, CA, USA) and eluted via 1.5% agarose electrophoresis. The Quanti-Fluor™-St (Promega, Madison, WI, USA) blue fluorescence quantitative system was used to detect PCR products. Following Illumina’s standard protocols, the amplicons that had been purified were mixed equimolarly and then subjected to paired-end sequencing (2 × 300) [36]. The sequencing procedures were conducted by the Shanghai Majorbio Bio-pharm Technology Company in Shanghai, China.

The data were then processed, using Trimmomatic software (v0.36) for quality checking and Flash software (v1.2.7) for splicing. After trimming the barcode sequences and adapter regions, quality-filtering criteria were applied, which included a minimum of a twenty quality score of the sequence reads, no truncated reads shorter than 50 bp, a mismatch ratio between overlaps of less than 0.2, an overlap length greater than 10 bp, the exclusion of reads that could not be assembled, the generation of two primer base mismatches, and the discarding of reads with unclear characters. Additional denoising was performed inside each sample using a preclustering technique, followed by an operational taxonomic unit (OTU) clustering at a 97% pairwise identity with UPARSE (v7.0, http://www.drive5.com/uparse/, accessed on 11 December 2023).

The RDP Classifier method and the UNITE database Release 8, (https://unite.ut.ee/, accessed on 11 December 2023) were used to taxonomically classify representative sequences for each OTU. The Mothur program (1.30.1) [37] was used to calculate species richness and diversity indices, such as the Shannon, Simpson, Chao1, and ACE indices. This thorough approach provided essential insights into the culture-independent endophytic fungal community, bolstering the scientific rigor and data reliability of this investigation.

### 2.5. Assessment of Growth-Promoting Traits

Specific culturable EFs were examined for indole-3-acetic acid (IAA) production, siderophore synthesis, and phosphate solubilization to assess their growth-promoting properties. The EFs were inoculated into specialized liquid media and incubated at 28 °C for 10 days at 125 rpm. The IAA concentration was measured using a Salkowski reagent [38]. EF isolates were inoculated in potato dextrose broth (PDB) supplemented with 0.5 mg/mL L-tryptophan. Uninoculated tryptophan-enriched broth mixed with Salkowski reagent (1:1) served as the control. Iron-free Czapek–Dox broth was used to identify siderophore synthesis by EF, while siderophore production was estimated by a chrome azurol sulfonate (CAS) assay [38]. In addition, the ability to solubilize phosphate was assessed using the methodology described by Attia et al. [39]. EFs were grown on National Botanical Research Institute Phostphal Growth Medium (NBRIP) agar plates at 30 °C for 5 days. After incubation, a clear halo zone around the fungal colonies initially indicated phosphate solubilization. The isolates that were positive for phosphate solubilization were further investigated for quantitative analysis in NBRIP broth. The amount of soluble phosphate (mg/L) was assessed using a UV-1800PC UV–Vis spectrophotometer (MAPADA, Shanghai, China). Importantly, all experiments were conducted in triplicate to confirm the reproducibility and precision of the data.

### 2.6. Pot Experiments

Corn seeds (*Zea mays* cultivar Huidan 4#) were sterilized as described above for pot experiments to determine the efficiency of EFs, which have remarkable growth-promoting properties. The selected EFs were cultured on PDA media at 28 °C for 4–7 days. Then, 500 mg of mycelia was collected, chopped into small (1–3 mm) pieces with sterile scissors, and suspended in 300 mL of sterile distilled water.

The sterilized seeds were randomly divided into two portions. Portion I was immersed in the fungal suspension for one hour to facilitate fungal inoculation (EF+). Portion II was the control and was immersed in sterile water (EF-) for one hour. Then, EF+ and EF- seeds were planted in pots with a mixture of autoclaved field soil and perlite (7:3 *v*/*v*) containing 15 mg/kg CdCl_2_. Each treatment had nine replicates, with six seeds planted in each pot. Three plants were left in each pot when the first pair of genuine leaves appeared [40]. The pots were placed randomly and kept under artificial plant lighting, following a light/dark cycle of 16/8 h. Plants were irrigated with sterilized water every 6–7 days as needed. On the tenth day, the plants were subjected to a second round of vaccination by spraying with mycelium suspension or sterile water. The plant height, root length, and dry biomass were measured after a cultivation period of 28 days as part of the assessment procedure.

### 2.7. Statistical Analysis

The statistical analyses were performed using SPSS software version 11.5. A multiple-range test, called Duncan’s test, was used to evaluate differences between the inoculated endophytes, with a significance level set at *p* < 0.05. Venn diagrams were constructed using TBtools software (v1.112) to facilitate visualization. The collected results are presented as the mean ± standard.

## 3. Results

### 3.1. Culture-Dependent Method

A total of 677 fungal isolates were obtained from 1125 corn grains collected across different sites in the culture-dependent analysis, allowing for a distinction between the three sites. The distribution of these EF isolates varied between the locations; 234 isolates were obtained from site AK, 283 from site SD, and 170 from site DL. The EFs’ colonization rates (CRs) of the corn grains from the AK, SD, and DL sites were 45.33%, 49.07%, and 29.33%, respectively. This difference was statistically significant (*p* < 0.05, *t*-test).

**Figure 1:**
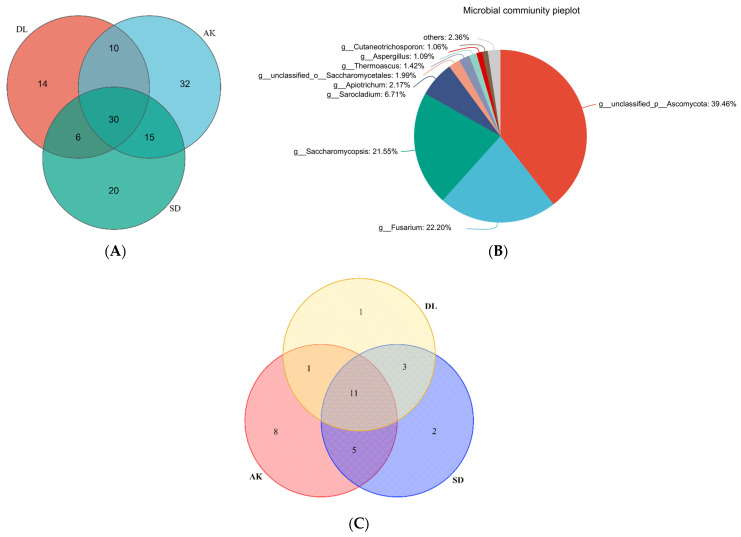
The unique and shared corn grain EFs from different HM-contaminated soils at the genus level. (**A**,**B**) Culture-independent EF; (**C**) culture-dependent EF.

The isolates were identified into thirty-one genera based on morphological characteristics and ITS sequencing analysis. Eleven genera (35.48%) were present at all three sites. In contrast, some genera were specific to the site, of which eight genera (25.8%) were found only at site AK, two (6.45%) were found at site SD, and one (3.22%) was found at site DL. Furthermore, AK and SD shared five genera (16.12%), AK and DL shared one genus (3.22%), and DL and AK shared three genera (9.67%) (Figure 1C).

**Figure 2:**
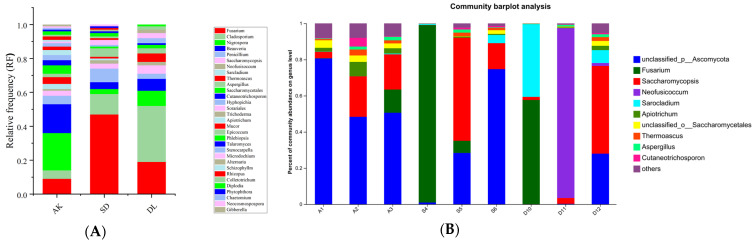
The relative abundance of corn grain EFs at the genus level. (**A**) Culture-dependent EF; (**B**) culture-independent EF. The fungi with a relative abundance less than 0.1% were grouped as ‘others’. A1–A3 = AK; S4–S5 = SD; D10–D12 = DL.

Five genera emerged as prevalent across the samples: *Fusarium*, *Cladosporium*, *Nigrospora*, *Beauveria*, and *Penicillium*, with relative frequencies of 23.34%, 21.13%, 11.23%, 6.21%, and 5.22%, respectively. Nevertheless, there were slight variations in dominance patterns across the different sites. In AK, the most common genera were *Nigrospora* (22.65%), *Beauveria* (17.8%), and *Fusarium* (9.65%). In the SD site, the predominant genera were *Fusarium* (47.15%), *Cladosporium* (12.25%), and *Penicillium* (8.69%), while in the DL site, the predominant genera were *Cladosporium* (33.24%) and *Fusarium* (19.84%) (Figure 2A).

The diversity indices provided a clearer understanding of the EF population differences between the three locations. Table 2 shows all the diversity indices of the three sites. AK’s was greater than SD’s and DL’s. The rDNA ITS sequences of the EFs analyzed for molecular identification in this study have been deposited in GenBank (the accession numbers are PP474095, PP474096, PP474097, PP474098, PP474099, PP474100, PP474101, PP474102, PP474103, PP474104, PP474105, PP474106, PP474107, PP474108, PP474109, PP474110, PP474111, PP474112, PP474113, and PP474114).

### 3.2. Culture-Independent Endophytic Fungal Community

**Figure 3:**
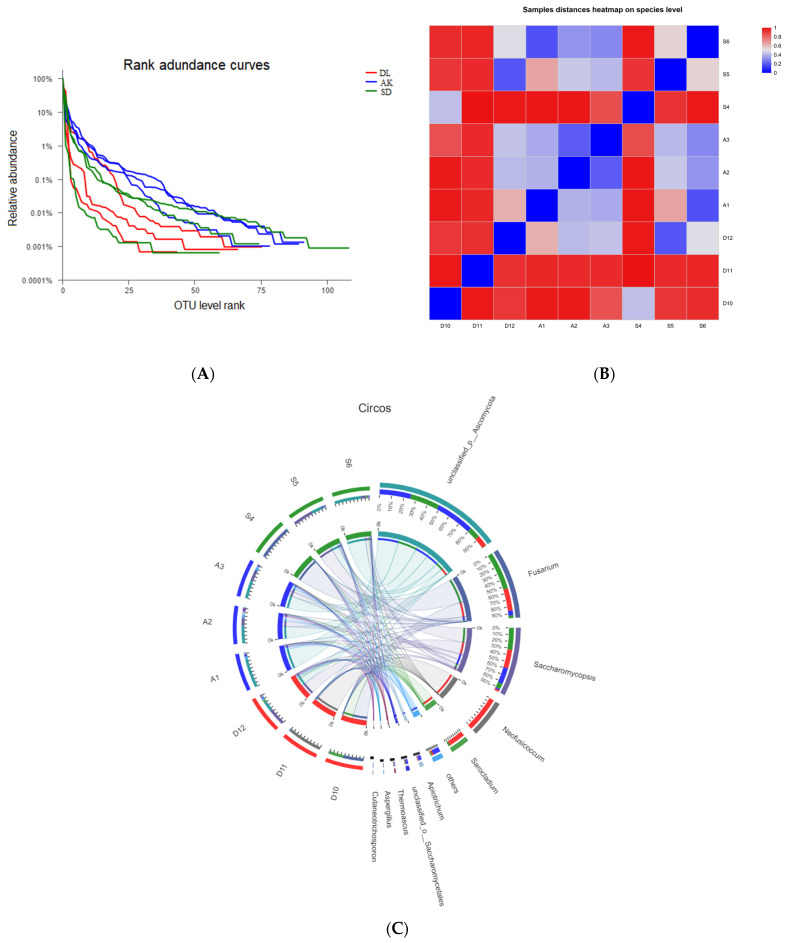
The diversity of culture-independent corn grain EFs between contaminated and non-contaminated sites. (**A**) The rank curve is based on OTU abundance. (**B**) The beta diversity heatmap is based on the unweighted UniFrace distance. (**C**) Circos graph. Different colors indicate differences in species diversity among the samples. A1–A3 = AK; S4–S5 = SD; D10–D12 = DL.

A dataset of 1,001,229 filtered, high-quality, and classified unique fungal ITS1F_ITS2R gene tags with an average length of 258 bp was developed (Table 3). These EF sequences were grouped into 239 OTUs classified into 127 genera. Among them, thirty genera were present at all three sites. AK shared fifteen genera with SD and ten genera with DL, while DL shared six genera with SD. Each site also had a unique set of genera: thirty-two genera in AK, twenty in SD, and fourteen in DL (Figure 1B). This highlights that specific environmental conditions influence the unique fungal diversity at each site. The OTU rank abundance curve confirmed that EFs from HM-contaminated areas (AK and SD) had a greater diversity and abundance than those from the non-contaminated area DL (Figure 3A). This pattern indicates that HM contamination could impact the composition of the fungal community, favoring fungal species or strains that can tolerate or adapt to HM-induced stress.

The OTUs were methodically classified into a hierarchical structure that included 10 phyla, 25 classes, 48 orders, 93 families, 127 genera, and 165 species. The phylum *Ascomycota* was the most prevalent in all the samples, indicating its dominant position in the fungal community. *Basidiomycota*, *Mortierellomycota*, and *Chytridiomycota* followed. The genera-unclassified *Ascomycota*, *Fusarium*, *Saccharomycopsis*, *Neofusicoccum*, and *Sarocladium* were abundant, demonstrating their significant presence in the studied settings.

The study’s computational analysis of α diversity, using a threshold of 0.03 distance units for OTUs, allowed for a thorough evaluation of the abundance and variety of species within the samples (Table 3). The Chao1 and Ace metrics supplied estimates for the minimum number of OTUs, while the inverse Simpson and Shannon diversity indices provided information about the community’s richness. AK had a significantly greater Shannon index (1.8) than SD (0.8) and DL (0.9).

**Figure 4:**
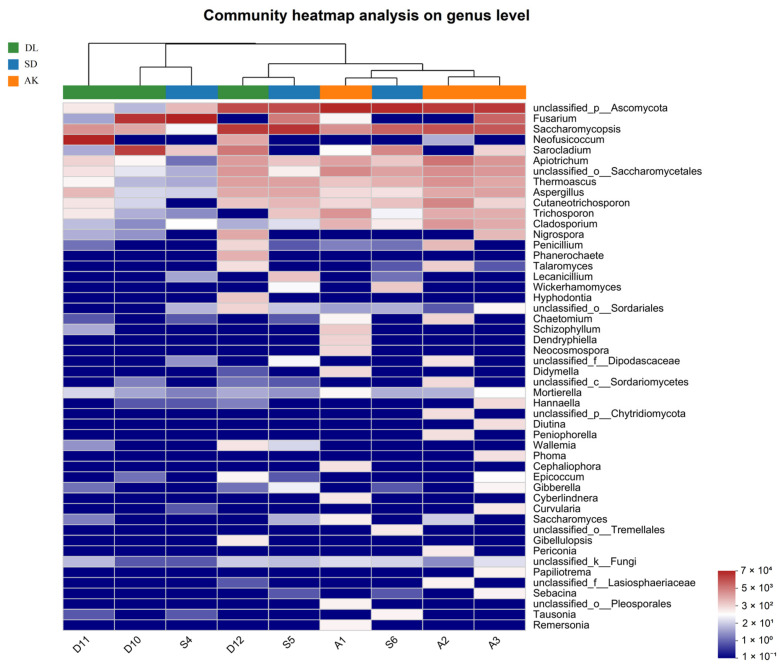
Heatmaps of the relative abundance of culture-independent EFs at the genus level, based on an analysis of corn grains of the 50 most abundant genera. Different colors indicate differences in the relative abundance (log10) of the taxa in the samples (red indicates a high relative abundance).

A beta diversity analysis revealed considerable differences in the diversity of EF species between contaminated areas (AK and SD) and non-contaminated area (DL) (Figure 3B). Correspondingly, the differences were visually depicted and verified using a Circos plot and a heatmap (Figure 3C and Figure 4). This thorough assessment elucidates the influence of environmental elements, such as HM, on the variety and spread of fungal communities within the tissues of corn grains. This study provides valuable knowledge about the ability of these fungal species to withstand and adjust to different ecological conditions.

### 3.3. PGP Traits of Endophytic Fungi

Sixty EF isolates were randomly selected from twenty-two genera to assess PGP traits. The results showed that 70% (forty-two isolates) of the tested isolates demonstrated phosphorus solubilization, IAA production, or siderophore production activity, while only 11.67% (seven isolates) exhibited all three PGP activities.

**Figure 5:**
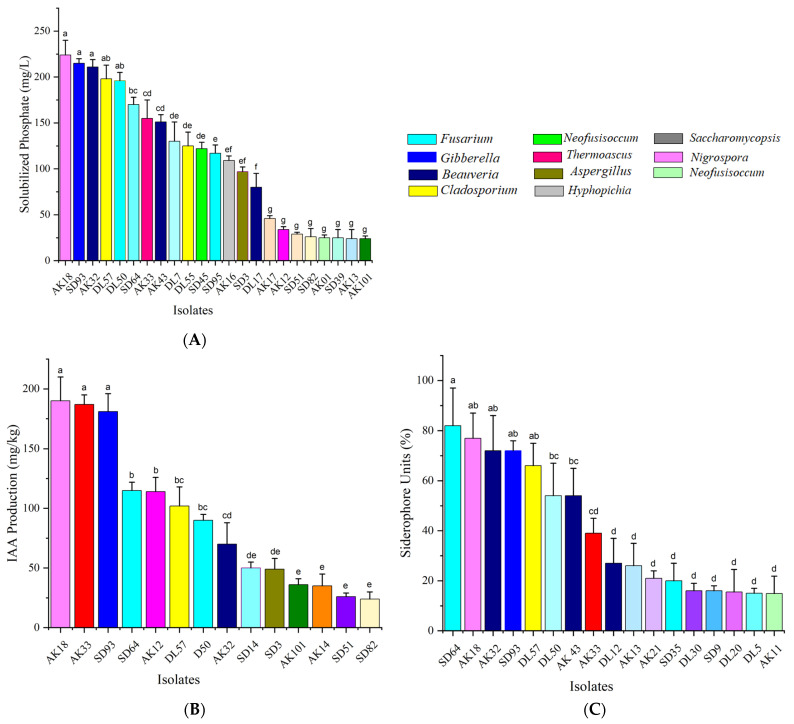
(**A**) Phosphorus solubilization; (**B**) IAA production; (**C**) siderophore production of corn grains EFs. Different colors represent distinct genera, while identical colors denote the same genus. Different letters in each column denote that mean values are significantly different (*p* < 0.05), means *±* SDs (*n* = 3).

Phosphorus solubilization was detected in 38.33% (twenty-three isolates) of the examined endophytes from eleven genera. The phosphorus solubilized by these isolates exhibited a wide range, spanning from 24.39 to 223.89 mg/L. The AK18 isolate, which belongs to the genus *Nigrospora*, was the most effective at solubilizing phosphorus, with a concentration of 223.89 mg/L. It was closely followed by isolates SD93 (*Gibberella*), with a concentration of 214.78 mg/L; AK32 (*Beauveria*), with a concentration of 210.8 mg/L; and DL57 (*Cladosporium*), with a concentration of 197.67 mg/L (Figure 5C).

IAA synthesis capacity, a critical factor affecting plant growth and development, was found in 23.33% (fourteen isolates) of the seven genera. The production levels of IAA varied, with that of the isolate AK18 (*Nigrospora*) being the highest at 189.65 mg/L. Additional noteworthy isolates were AK33 (*Thermoascus*), SD93 (*Gibberella*), and SD64 (*Fusarium*), which exhibited IAA production at concentrations of 186.61 mg/L, 180.28 mg/L, and 89.77 mg/L, respectively (Figure 5A).

Siderophore synthesis, a characteristic that helps plants acquire iron, was found in 28.33% (seventeen isolates) of the EFs studied. The isolates exhibited siderophore units (SUs) of more than 10%, ranging from 14.83% to 81.66%. Significantly, seven isolates had SU production rates of 50% or more. Among them, isolate SD64 (*Fusarium*) exhibited the highest percentage at 81.78%, while AK18 (*Nigrospora*) and AK32 (*Beauveria*) obtained percentages of 76.71% and 71.64% SU, respectively (Figure 5B).

The data indicate that endophytic isolates from areas exposed to high levels of HM pollution, notably AK and SD, exhibited more significant PGP tendencies than did those from the nonpolluted DL site.

### 3.4. Pot Experiments

**Figure 6:**
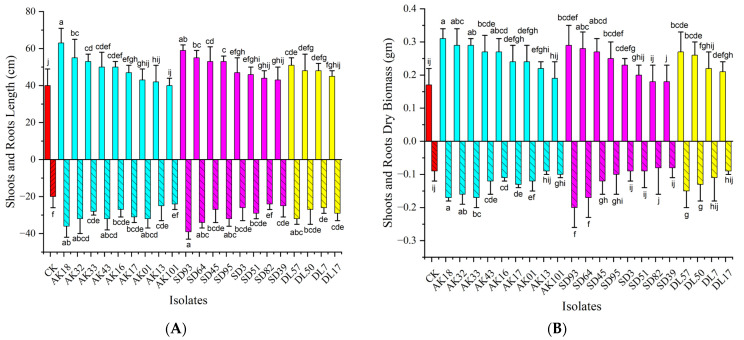
(**A**) Relative length and (**B**) relative biomass of shoots and roots of *Zea mays* inoculated with different isolates. The red bars represent CK, while the cyan, magenta, and yellow bars represent AK, SD, and DL respectively. Different letters in each column denote that mean values are significantly different (*p* < 0.05), means *±* SDs (*n* = 9).

Twenty-one isolates with better PGP traits were selected for pot experiments. The results showed that all the selected isolates significantly enhanced the growth of maize. Strains derived from HM-contaminated sites, including AK18 (*Nigrospora*), AK32 (*Beauveria*), SD93 (*Gibberellia*), and SD64 (*Fusarium*), had notable effects on enhancing the dry biomass of both the shoots and roots of maize (*p* < 0.05). A substantial difference was detected between the experimental group (EF+) and the control group (EF-) (Figure 6A). This contrast highlights the effectiveness of the fungal isolates in reducing the harmful effects of Cd and enhancing plant growth. Among the tested isolates, AK18 (*Nigrospora*) and SD64 (*Fusarium*) were the most effective, exhibiting the most substantial growth-promoting effect compared to the control group (EF-).

## 4. Discussion

The major and emerging threat to modern humanity is the contamination of arable land with HMs from different anthropogenic activities [41]. As a result of contamination, several resistant microbial species have become abundant. These microbes, either endophytic or rhizospheric, then have the capability to help the host plant to withstand harsh conditions, while ensuring normal host health. Among these, endophytes can enhance crop resilience to HM contamination by promoting plant growth, aiding adaptability to stressful environments, and increasing tolerance to HMs through the secretion of secondary metabolites to regulate plant responses [42,43]. The present study showed that areas with HM contamination (AK and SD) exhibited a greater diversity and abundance of EFs than non-contaminated areas (DL). This is because plants under stress release certain chemicals that attract beneficial microbes [44]. These chemo-attractants draw in microbial species capable of promoting plant growth and stress resilience, becoming part of the plant microbiome [45]. A similar study was also carried out by Passarini et al. [46], which showed that HMs, such as copper (Cu), play a crucial role in shaping fungal diversity in contaminated environments. Previous research has also indicated that soil HM contamination levels influence the diversity and tolerance of EFs associated with hyperaccumulators such as *Dysphania ambrosioides* [47]. In contrast, Huang et al. [48] reported that HM contamination reduced the diversity and richness of seed endophytic communities, mentioning that susceptible microbes are removed by the toxicity of HMs and resistant microbial species thrive in harsh conditions and become dominant. These findings suggest that HM contamination can lead to the vanishing of important microbial flora and the microbiome narrowness of seed EFs, thereby affecting plant responses to HM stress.

Plants tend to adapt to harsh environmental conditions by the release of stress metabolites and chemo-attractants. Stress-related metabolites help the plant to cope the harsh conditions [49,50]. However, chemo-attractants help the plant to attract the best tolerant and resistant microbes. These microbes help the host by the release of plant growth hormones, which further boosts the host defense mechanisms to cope with stress and enhances their growth attributes [45]. This was further confirmed in our study, which found that the majority of the dominant fungal endophytes in the study have multiple PGP traits like the production of phytohormones, solubilizing insoluble phosphate, and the production of siderophores that not only inhibit pathogens but also improve the host agronomic attributes. EFs from contaminated environments displayed notably greater PGP traits than those from non-contaminated areas (Figure 5). This is due to the fact that microbes also have different strategies to cope with HM stress by releasing growth hormones, metalloprotein, siderophores, chelating proteins, stress hormones, and metabolites that aid them to grow normally. Sometimes, microbes use these metals as Redox agents to drive their metabolic processes [51]. The higher PGP traits also show that the microbes tend to increase the number of their cells, which is a strategy to divide the stress and effectively handle toxic effects [52]. A similar pattern was also recorded in the current study, where EFs demonstrated an enhanced ability to improve the growth attributes of the host plant in Cd-supplemented soil conditions (Figure 6). This study was further supported by the findings of Gowtham et al. [53] and Zheng et al. [54], who described that EF enhances the host plant adaptation to HM stress through multiple mechanisms, i.e., phytohormone production, the synthesis of lower-molecular-weight protein for osmotic regulation, carbon and nitrogen metabolism, immune activity enhancement, and antioxidant enzyme production. Additionally, Cui et al. [55] reported that EFs improved detoxification and HM emission capacity by producing essential components such as iron carriers, metallothionein, and 1-aminocyclopropane-1-carboxylic acid deaminase.

Previous studies have shown that microbial endophytes directly promote plant growth through phosphorus mobilization [56] and plant hormone production [39], and the production increases to some extent as the contaminant level increases to cope with the conditions of HM stress. Recent studies also recorded that the increase in HM in the medium showed a positive correlation with phytohormone and metabolite production in *Pantoea conspicua* and *Aspergillus niger* [57]. Additionally, these endophytes can provide essential iron for various cellular functions [58]. In our study, 70% of the tested isolates exhibited phosphorus solubilization, IAA production, or siderophore production, with 11.67% possessing all three PGP activities (Figure 5). Jain et al. [59] found similar results with endophytes of *Arnebia euchroma*, where many EFs exhibited one or more critical PGP traits. Consistent with these findings, the isolates AK18 and SD64 from HM-contaminated sites demonstrated a higher PGP activity and significantly increased plant growth in pot experiments (Figure 6). These isolates were identified as *Nigrospora* and *Fusarium* spp. and were found to be the most dominant culturable EFs in HM-contaminated sites in the present study, while *Cladosporium* was prevalent in non-contaminated areas (Figure S1). Nguyen and Phan [60] highlighted the prevalence of *Nigrospora* in plants growing on HM-contaminated soils, demonstrating its resistance to Cd. Goryluk-Salmonowicz et al. [61] also studied the presence of *Nigrospora* as a Cd-resistant fungus in roots from slag heaps and reported that *Nigrospora* is well adapted to harsh environments and plays a crucial role in plant adaptation to HM stress.

*Fusarium* spp. are recently reported to have a higher plant growth-promoting and heavy metal remediation and stress alleviation potential [62,63,64]. Their abundance in seeds from contaminated environments could indicate potential adaptation to high levels of HM contamination; HM contamination is known to impose selective pressures on microbial communities, leading to the emergence of metal-tolerant or metal-utilizing strains. Studies have shown that *Fusarium* species such as *F. oxysporum* and *F. verticillioides* can tolerate Cd contamination and play a role in reducing Cd content in plants; these mechanisms include the production of metallothioneins and other metal-binding proteins, which can sequester and detoxify HMs, thereby reducing their toxicity [65]. Additionally, the occurrence of *Fusarium* species such as *F. verticillioides* and *F. graminearum* has been linked to the production of mycotoxins in cereals, highlighting their ability to thrive in contaminated environments [66]. Research has demonstrated that *Fusarium* species are commonly found in various seeds and grains, with different species dominating on different substrates, showing their adaptability to diverse conditions, including HM-contaminated soils [67,68]. They have the ability to promote seed germination by the production of Gas, which further have a link with starch and sugar metabolism to provide glucose as food to the newly growing embryo [69]. In later stages, as mentioned by several recent studies, different *Fusarium* strains perform multifold functions in plant growth and development and improved resilience by modulating phytohormone and stress-related metabolite production and stress mitigation [70].

## 5. Conclusions

From the current study and evidence, it has been revealed that the contamination of arable land is a hot and emerging threat to agriculture. Such contamination nurtures an abundance of resistant EFs, which perform multifold functions, enhancing plant resilience to HM stress. These selected EFs in the study tend to promote plant growth by producing phytohormones, solubilizing phosphate and secreting siderophores. In HM-contaminated areas, EFs displayed greater PGP traits compared to those from non-contaminated areas, aiding plants in coping with stress. Notably, *Nigrospora* and *Fusarium* spp. from contaminated sites showed high PGP activity and significantly improved plant growth. These fungi tolerate and detoxify HMs through metallothionein production and other mechanisms, enhancing plant adaptation to HM stress. This study underscores the potential of utilizing EFs to boost crop resilience and productivity in HM-contaminated environments.

## Figures and Tables

**Table 1:** Details of the corn collection sites.

Location	Altitude (m)	UTM Coordinates	Cd (mg/kg)	Pb (mg/kg)	Zn (mg/kg)
DL	1975	25°36′23″ N 100°16′03″ E	0.51	29.18	71.07
SD	2130	26°28′16″ N 103°37′33″ E	9.47	356.5	993.8
AK	2120	26°34′05″ N 103°37′06″ E	15.07	781.66	1677

**Table 2:** Alpha diversity indices of culturable EFs in corn grains.

Corn Collection Sites	Diversity Indices of EF				
	Taxa (S)	Shannon	Evenness	Chao1	Simpson
AK	16.67 ± 2.52	2.50 ± 0.05	0.89 ± 0.04	19.29 ± 1.26	0.92 ± 0.03
SD	12.00 ± 3	1.85 ± 0.09	0.76 ± 0.05	12.25 ± 3.38	0.75 ± 0.01
DL	10.67 ± 2.08	2.04 ± 0.15	0.87 ± 0.01	11.00 ± 2.65	0.84 ± 0.01

**Table 3:** The number of OTUs and alpha diversity indices of culture-independent EFs of corn grains (distance < 0.03).

Seed Collection Sites	Number of Sequences	OTU	α-Diversity			
			Shannon	Simpson	Ace	Chao
AK	65,310 ± 11,234	93	1.8 ± 0.40	0.34 ± 0.15	95.2 ± 2.85	96.67 ± 0.32
SD	118,484 ± 36,014	84	0.84 ± 0.49	0.64 ± 0.28	98.93 ± 20.6	94.57 ± 19.6
DL	125,568 ± 21,512	62	0.92 ± 0.66	0.57 ± 0.29	94.33 ± 12.9	76.67 ± 14.3

## Data Availability

The original contributions presented in the study are included in the article/Supplementary Materials, further inquiries can be directed to the corresponding author.

## Supplementary Materials

The following supporting information can be downloaded at: https://www.mdpi.com/article/10.3390/jof10080581/s1, Figure S1: Dominant fungal endophytes (EFs) from Heavy metal (HM) contaminated sites (AK and SD) and Non-contaminated Site (DL).
