# Supplementary material for: The Mechanisms of Cadmium Stress Mitigation by Fungal Endophytes from Maize Grains
Source: J Fungi (Basel). 2024 Aug 16;10(8):581. doi: 10.3390/jof10080581 (PMC11355786; doi:10.3390/jof10080581)
Supplement: Supplementary file 1 [file jof-10-00581-s001.zip › jof-3105669-supplementary.pdf]

Supplementary File

HM-contaminated site (AK)

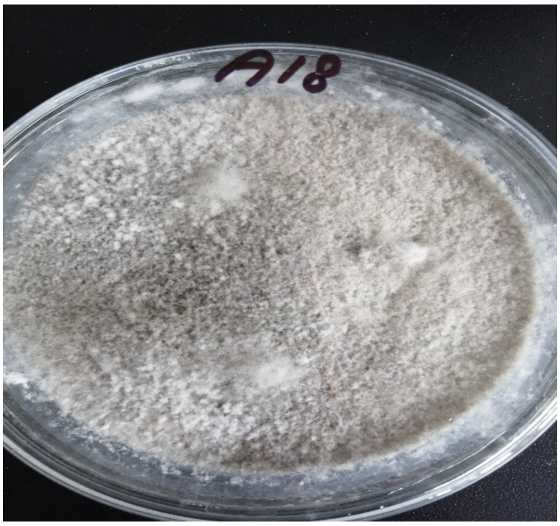

Nigrospora spp.

HM-contaminated site (SD)

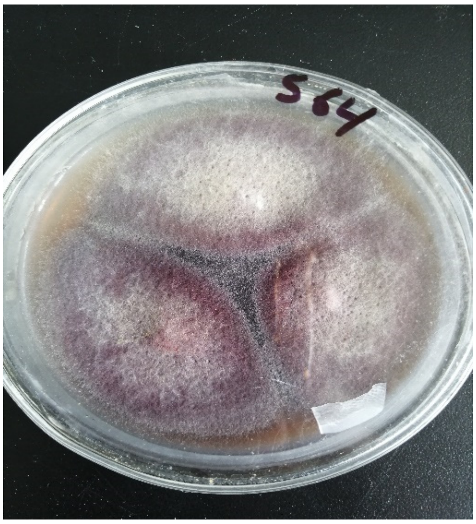

Fusarium spp.

Non-contaminated site (DL)

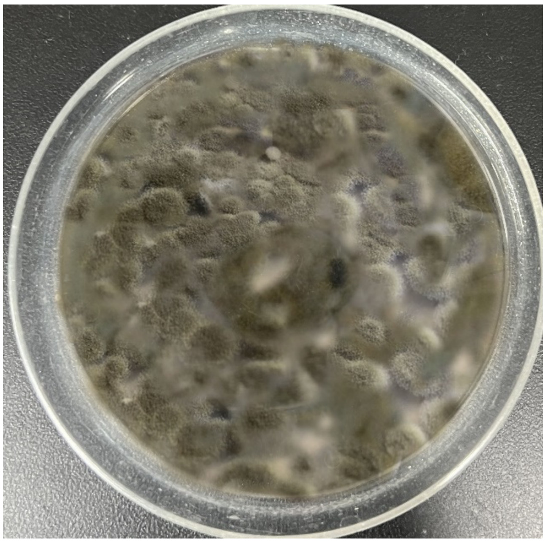

Cladosporium spp.

Figure S1. Dominant fungal endophytes (EFs) from Heavy metal (HM) contaminated sites (AK and SD) and Non-contaminated Site (DL).
